# Samuel Bowles and Dr. Holland

**Published:** 1881-12

**Authors:** Edward Eggleston


### SAMUEL BOWLES AND DR. HOLLAND.

That was a rare conjunction which brought together on the same paper, in a small inland town, two men of such journalistic ability as Holland and Bowles. On that side of journalism which affects public life, Samuel Bowles was one of the greatest of his class. Editing a paper that could never be other than provincial, his rare insight and foresight, his unpartisan frankness, his rugged and even rude integrity, made the opinions of his paper more valuable, and its adverse judgments more feared, than those of any other journal in the nation. Greeley and Raymond were great partisan advocates, but Bowles was a journalistic day of judgment. His masterpiece of wisdom in selecting his lieutenants was his hitting upon Doctor Holland, whose gifts were of a kind precisely opposite to his own. Mr. Bowles' attention was absorbed by public questions and the business management of the paper; Holland, though writing on national topics, had small relish for politics. He was the most popular and effective preacher of social and domestic moralities in his age; the oracle of the active and ambitious young man; of the susceptible and enthusiastic young woman; the guide, philosopher and schoolmaster of humanity at large, touching all questions of life and character. If Bowles made the *Republican* esteemed and feared in Massachusetts and the nation, Holland made it loved in ten thousand homes. Wanting either of these men, the paper must have failed to become what it did.—*Edward Eggleston in The Century for December.*

